# Analysis of Selected Eye Disorders in a Group of Predisposed Breeds of Dogs: Molecular Diagnostics of Collie Eye Anomaly and Progressive Retinal Atrophy

**DOI:** 10.3390/genes16050474

**Published:** 2025-04-23

**Authors:** Jaroslav Bučan, Beáta Holečková, Martina Galdíková, Jana Halušková, Viera Schwarzbacherová

**Affiliations:** Department of Biology and Physiology, University of Veterinary Medicine and Pharmacy in Košice, Komenského 73, 04181 Košice, Slovakia; beata.holeckova@uvlf.sk (B.H.); martina.galdikova@uvlf.sk (M.G.); jana.haluskova@uvlf.sk (J.H.); viera.schwarzbacherova@uvlf.sk (V.S.)

**Keywords:** hereditary eye disorders, dog, *CEA* gene, *PDE6A* gene, mutation, PCR, RT-PCR

## Abstract

Background: Two hereditary eye disorders that are frequently observed in Collies and related breeds are Collie Eye Anomaly (CEA) and Progressive Retinal Atrophy (PRA). The main symptom of CEA is choroidal hypoplasia. It is associated with a 7.8 kb deletion in intron 4 of the *NHEJ1* gene located on chromosome CFA7. Rod–cone dysplasia 3 (RCD3), an early-onset form of PRA, is associated with mutations in the *PDE6A* gene. Methods: Molecular diagnostic techniques were used in this study to identify genetic mutations linked to CEA and RCD3-type PRA in a subset of dog breeds. Australian Shepherds (n = 29), Border Collies (n = 9), Longhaired Collies (n = 27), and Shetland Sheepdogs (n = 10) provided a total of 75 DNA samples. Samples were collected by buccal swab or blood draw, and PCR and real-time PCR methods were used for processing. Results: Of the dogs in the studied breeds, 31 had the *NHEJ1* gene mutation linked to CEA. Among these, 15 were homozygous recessive (affected), while 16 were heterozygous (carriers). None of the samples had any mutations in the *PDE6A* gene associated with RCD3-type PRA. Conclusions: Effective identification of carriers and affected individuals for CEA was made possible by PCR-based genetic testing, confirming its value in early diagnosis and breed control. Although the RCD3 form of PRA has not been previously reported in Collies or Australian Shepherds, it was included in our analysis due to the genetic relatedness among herding breeds and the potential presence of undetected carriers resulting from historical crossbreeding.

## 1. Introduction

Dogs represent a suitable animal model for studying diseases. According to the OMIA (Online Mendelian Inheritance in Animals) database, 969 genetic diseases are described in dog breeds, and 626 of them were indicated as potential models for humans [1]. Moreover, eye diseases in dogs are naturally developed, so they represent a spontaneous model for numerous human retinal ones [2]. Other advantages of dogs as an animal model are faster development of eye disease because of shorter life spans, good medical care in developed countries, and high numbers of offspring with the access of many individuals and pedigrees for analysis. Finally, natural animal models such as dogs are important for the development of future treatments/therapeutic strategies, including gene therapies in humans [3].

The inherited forms of eye diseases represent a large group of disorders that significantly reduce the vision of dogs. They are one of the best described and characterized disorders due to the easy access for examination [4]. Although some diseases can only be correctly diagnosed in a short period of time after being born, like Collie Eye Anomaly. For this reason, genetic testing is very important for the correct determination of health status. Knowing the exact genotype of individual dogs has enabled the option to eliminate carriers from breeding and ensure healthier offspring [5]. The importance of testing is also confirmed by the fact that hereditary retinal disorders are one of the major causes of blindness in the world [6].

Collie Eye Anomaly (CEA) is a hereditary ocular disorder mainly affecting Collies and other related dog breeds, such as Australian Shepherd dogs. The main characteristic symptom is regional hypoplasia of the highly vascularized layer of the eye (choroid), which supplies blood and nutrients to the retina [7]. This abnormality can be identified through ophthalmoscopic examination between 6 and 12 weeks of age, a time frame known as the diagnostic window, when the pigmentation of the eye’s fundus is not yet fully developed. Scleral defects characterized by colobomatous lesions can also appear in or around the optic nerve head, often presenting as pits in the nearby fundus [8]. The disease can be complicated by secondary retinal detachment and intraocular hemorrhage. CEA affects both eyes, but the lesions are rarely symmetrical and of the same severity [9]. Clinically, choroidal hypoplasia does not appear to affect vision, while large optic disk coloboma can reduce it. Blindness can be caused by retinal detachment and intraocular hemorrhage, even though bilateral blindness rarely occurs [10]. This condition is linked to a 7.8 kb deletion within intron 4 of the canine *NHEJ1* gene, located on chromosome CFA7. The deletion has been identified in sheep-herding breeds related to the Collie line, as well as in other breeds such as the Nova Scotia Duck Tolling Retriever and the Longhaired Whippet [7]. A genetic test utilizing this association is capable of identifying three distinct genotypes: clear (normal), carrier, and affected [11].

Progressive Retinal Atrophy (PRA) represents a group of inherited retinal degenerative diseases that affect humans and various animals, including studied species. This hereditary disorder leads to gradual vision loss and ultimately blindness. One specific type, known as RCD3 (rod–cone dysplasia 3), is associated with a mutation in the *PDE6A* gene, which encodes the α subunit of the cGMP-phosphodiesterase enzyme [12]. This enzyme plays a critical role in the phototransduction cascade within rod photoreceptor cells, and its dysfunction can lead to the degeneration and death of these cells. The *PDE6A* gene mutation that causes RCD3-type Progressive Retinal Atrophy in dogs has been well characterized. The condition serves as a valuable model for understanding the underlying mechanisms of retinal degeneration and exploring potential therapeutic interventions [12,13,14,15]. Dogs affected by RCD3-type Progressive Retinal Atrophy typically experience a rapid and complete loss of rod photoreceptors, causing night blindness and, at last, total blindness by around one year of age. The condition is inherited in an autosomal recessive manner [14]. At the molecular level, the *PDE6A* mutation in RCD3 leads to a complete absence of rod phosphodiesterase activity, resulting in the accumulation of cyclic GMP (cGMP) in rod photoreceptor cells. The *PDE6A*-mutant dog model has been extensively studied as a valuable large animal model for understanding the underlying mechanisms of retinal degeneration and exploring potential therapeutic interventions to preserve or restore vision in individuals affected by similar forms of retinitis pigmentosa [12]. Cyclic GMP (cGMP) is a crucial signaling molecule in the visual transduction pathway, where it plays a central role in regulating the activity of ion channels and mediating the response of photoreceptor cells to light [12,15].

Genotyping of dogs for these diseases is very important due to their nature. CEA is known for the variable expression and pleomorphism in the affected individuals. Sometimes clinical symptoms are mild, and affected animals can retain normal vision throughout their lives [7]. Another significant reason is the small diagnostic window during the few weeks after birth. Due to these reasons, genotyping is significant for correct determination. Genetic testing should be used to confirm ophthalmological findings.

Our study focused on the analysis of 75 samples collected from breeds with a predisposition to CEA. Despite the fact that neither Collies nor Australian Shepherds have been known to carry the RCD3 form of PRA, our analysis includes it because of the genetic similarities between herding breeds and the possibility of undetected carriers as a result of past crossbreeding. Examining this variant may also help explain cases of retinal degeneration with an unclear genetic origin and enable more extensive epidemiological surveillance. This is especially pertinent given that closely related breeds like the Cardigan Welsh Corgi have a well-documented *PDE6A* mutation that causes RCD3 [16]. The group of examined dogs consisted of 29 Australian Shepherds, 9 Border Collies, 27 Longhaired Collies, and 10 Shetland Sheepdogs, in which we focused on detecting these mutations using molecular–genetic methods.

## 2. Materials and Methods

In our study, we analyzed 75 samples collected from breeds in which hereditary eye diseases are expected to occur. Specifically, we sought to confirm or refute these mutations in 29 Australian Shepherds, 9 Border Collies, 27 longhaired Collies, and 10 Shetland Sheepdogs. Samples were collected during a 2-year period. Individuals were examined by local EESVO-certified specialists. The characteristic changes in ocular background for CEA were observed in some of the individuals in this study, mainly in the young dogs. PRA was not confirmed. To evaluate these findings, especially in dogs that were older and an ophthalmologic diagnosis was not possible due to pigmentation of the ocular background, samples were collected from all dogs for genetic testing.

### 2.1. Sample Collection and DNA Isolation

Blood collection or buccal swabbing were the two sampling techniques used to obtain biological material from the individuals being examined. Usually, from the cephalic vein, blood samples were extracted into the sterile tubes with the anticoagulant ethylenediaminetetraacetic acid (EDTA). Buccal swabs were carried out according to a standard protocol for collecting epithelial cells. Cytological brushes were used to obtain samples of oral mucosa, periodically alternating the direction of movement for five seconds on both sides. To guarantee a sufficient sample, two brushes were utilized. Before sampling, subjects had to refrain from eating and drinking for at least fifteen minutes in order to reduce contamination. The collection procedure adhered to the guidelines suggested by the Genomia Genetic Laboratory (Pilsen, Czech Republic), which uses this approach for a number of genetic tests, including CEA and PRA.

DNA isolation from blood samples was performed using the commercial ReliaPrep™ Blood gDNA Miniprep System (Promega, Madison, WI, USA). The same system was also used for buccal swab samples, but the isolation procedure was slightly modified to account for the unique properties of the sample. To be more precise, the water bath incubation period was prolonged from 10 to 30 min in order to guarantee total lysis of the epithelial cells. To assess the quality and concentration of nucleic acids, the extracted DNA samples were evaluated using the NanoPhotometer P300 (Implen, Munich, Germany). Test tubes were stored in a freezer at a temperature of −18 °C before the next step of the analysis.

### 2.2. Primers for CEA

Samples were analyzed for the presence of the mutation in the *NHEJ1* gene by two methods. The classical PCR method was used for the primary determination of the presence of the mutation. The set of primers (Eurofins Genomics, Ebersberg, Germany) described by Parker [17] was used (Table 1). The first set (*NHEJ1*-F17 5-TCTCACAGGCAGAAAGCTCA-3 and *NHEJ1*-R17 5-CCATTCATTCCTTTGCCAGT-3) was used for the intact part of the *NHEJ1* gene, and the second set (*NHEJ1*-F20 5-TGGGCTGGTGAACATTTGTA-3 and *NHEJ1*-R23 5-CCTTTTTGTTTGCCCTCAGA-3) was used for the mutated segment with deletion. The final concentration of the primers in the mixture was 100 pM. We used GoTaq^®^ G2 Hot Start Polymerase (Promega, Madison, WI, USA) with 5× Flexi Buffer according to recommended concentration and MgCl_2_ varying from 2 to 2.5 mM. Nuclease-free water (Promega, Madison, WI, USA) was added to the final volume of 25 µL. The mixture was run in a thermocycler. The PCR program consisted of several steps, starting with initial denaturation at 95 °C for 2 min, followed by 35 repetitions of second denaturation at 95 °C for 40 s, primer hybridization at 50 °C for 50 s, and elongation of the strand at 72 °C for 90 s. PCR was ended by final synthesis at 72 °C for 5 min.

### 2.3. Primers for PRA (RCD3)

Samples were analyzed for the presence of the mutation in gene *PDE6A* causing Progressive Retinal Atrophy type RCD3. For this analysis, we used a commercial primer set (Eurofins Genomics, Ebersberg, Germany), prepared according to Petersen–Jones [16] (Table 1). Two sets were designed to distinguish the mutated state of the gene from the wild type of the gene. The first pair of forward (SPJn-F, 5′-TCCCATTCAGGTCCCAGAA-3′) and reverse primer (SPJn-R, 5′-TGATGACCTCTGACCTCTG-3′) together yields a 212 bp amplicon that is only present in the wild type of the gene (AA). On the other hand, the second pair of forward (SPJm-F, 5′-TTCCCATTCAGGTCCCAGAC-3′) and reverse primer (SPJm-R, 5′-GATGACCTCTGACCTCTGAT-3′) form a 213 bp amplicon that can be produced only in the mutated gene in recessive homozygotes (aa). If there is amplification in both sets of primers, the individual is heterozygous in this gene and therefore is a latent carrier of the disease. Chemicals used for the reaction were the same as for the previous disease. PCR conditions for this disease were set according to Petersen–Jones [16] and consist of initial denaturation at 94 °C for 4 min, 30 repetitions of second denaturation at 94 °C for 30 s, and hybridization and elongation at 72 °C for 1 min. Hybridization was different for the wild and mutant types of the gene. For set of primers SPJn-F/SPJn-R, the temperature was 58 °C (1 min), and for the second set of primers, SPJm-F/SPJm-R, it was 61 °C (1 min). For this reaction, we used a Gradient Thermocycler Biometra TOne (Analytic Jena, Jena, Germany) to be able to set different hybridization temperatures. The final step was synthesis at 72 °C for 5 min. Amplicons from all standard PCR reactions were electrophoretically separated in 1.5% agarose gel. For visualization of bands under UV light, GelRed nucleic acid stain solution (Biotium, Fremont, CA, USA) was added to the gel at 1% concentration. Separation was performed in an electrophoretic bath Nanocell 100 (Biorad Laboratories, Hercules, CA, USA) in a solution of 1xTAE buffer (Serva, Heidelberg, Germany) using an electric source MS-MP300V (Major Science, Taoyuan City, Taiwan) at set conditions of electrical voltage of 75 V for duration of 60–65 min. In all runs we also used a 100 bp DNA Ladder solution (Thermo Fisher Scientific, Waltham, MA, USA) to determine the size of the PCR amplicon. After separation, the gel was photographed using a Transilluminator VWR Genoview (Avantor, Radnor, PA, USA), and results were subsequently evaluated.

### 2.4. RealTime PCR

For confirmation, all samples were reanalyzed by real-time PCR. Commercially available SYBR Green Master mix (Thermofisher, Waltham, MA, USA) was used. The 20 µL reaction mixture consisted of 10 µL of Mastermix, 1 µL of forward primer, 1 µL of reverse primer, and 8 µL of nuclease-free water. Primers were used according to Chang [8]. Forward primer (*NHEJ1*-FW, 5′-AGGGTTACCATTTGGGAACTGTCTT-3′) was designed for use with both reverse primers. The reverse primer RW for wild type (*NHEJ1*-RW, 5′-AGCTTCTGACAGGCCACAATTATCTA-3′) gives, together with the forward primer, a 120 bp amplicon that is only found in genetically healthy individuals (AA). The reverse primer RM for the mutant type of gene (*NHEJ1*-RM, 5′-ACCAATCATCATCCAGCCCAGCAGCATTTAA-3′) yields, together with the forward primer, an amplicon of size 68 bp, which is only found in diseased individuals with the mutation (aa). In the case of amplification of both primer pairs, the mutation is present on only one chromosome, and such an individual is a carrier of the disease (Aa). The reaction was carried out on a LightCycler^®^ 480 Instrument II (Roche, Bazilej, Switzerland). The amplification program used consisted of the activation of the PCR mix at 95 °C for 10 min, followed by 45 repetitions of the denaturation step at 95 °C for 15 s. Finaly, the annealing and extension step at 60 °C for 1 min ended the cycle.

## 3. Results

In our study, we analyzed 75 samples collected from various breeds that may be affected by hereditary eye disorders. The test group listed in Table 2 was composed of 29 Australian Shepherds, 9 Border Collies, 27 Longhaired Collies, and 10 Shetland Sheepdogs. Genotypes of all individuals were determined for both eye disorders.

### 3.1. Collie Eye Anomaly

**Figure 1:**
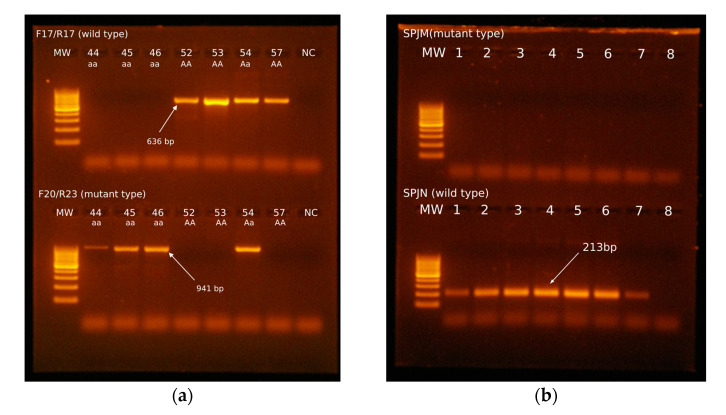
Electrophoretic analysis of PCR products with 100 bp molecular weight standard. (**a**) CEA analysis of selected samples. Samples 52, 53, and 57 (Longhaired Collies) yield only a 636 bp fragment using F17/R17 primers characteristic of the wild type of gene. These dogs are dominant homozygotes. In sample 54 (Longhaired Collie), there were amplicons of 636 bp and 941 bp that correspond with the heterozygous state of the gene *NHEJ1*. In samples 44, 45, and 46 (Longhaired Collies), there was amplification only with the second set of primers (F20/R23). This finding corresponds to the recessive state of the gene, characteristic of affected individuals. (**b**) Analysis of PRA-RCD3. Amplicons were present only with the first set of primers, SPJN, characteristic of the wild type of the gene *PDE6A*.

In 16 dogs (15 Longhaired Collies, sample Nos. 39–46, 51, 58, 60–63, and 65, and 1 Shetland Sheepdog, sample No. 75), the presence of a defect on the ocular background characteristic for CEA was ophthalmologically proven. This finding was confirmed by our analysis of the genotype, with amplification of a fragment of 941 bp using primers F20/R23 (Figure 1a). All dogs were recessive homozygous for the *NHEJ1* gene with deletion of a specific section of the sequence. Furthermore, sixteen dogs (nine Longhaired Collies, sample Nos. 47–50, 54–56, 59, and 64; one Border Collie, sample No. 35; two Australian Shepherds, sample Nos. 1 and 16; and four Shetland Sheepdogs, sample Nos. 66, 69, 72, and 74) with no changes observed in the ocular background were identified as latent carriers of the disease (heterozygous for the *NHEJ1* gene). In these samples, amplification occurs in both sets of primers, yielding a fragment of 636 bp and 941 bp. In other samples, amplification occurred only with the first set of primers (F17/R17), yielding a fragment of 636 bp that confirmed the dominant homozygous genotype characteristic for healthy individuals.

**Figure 2:**
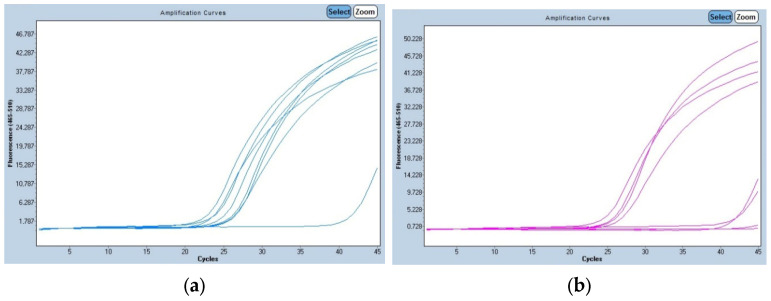
Real-time PCR results of CEA-affected individuals and carriers of the disease. (**a**) Fluorescence intensity of samples with the first set of primers, FW/RW, characteristic of healthy individuals. (**b**) Fluorescence intensity of samples with the second set of primers, FW/RM, characteristic of affected individuals.

To confirm the validity of the simple classical PCR method, we reanalyzed these samples by real-time PCR (Figure 2). We accepted the amplification of the 120 bp fragment for healthy individuals (AA) using the FW/RW set of primers, the 68 bp fragment for affected individuals (aa) using the FW/RM set of primers, and the amplification of both fragments for carriers (Aa) of the CEA. The real-time PCR confirmed the results of the conventional PCR reaction, with the same genotype for each sample. The fluorescence intensity went up approximately after 23 to 25 cycles.

**Figure 3:**
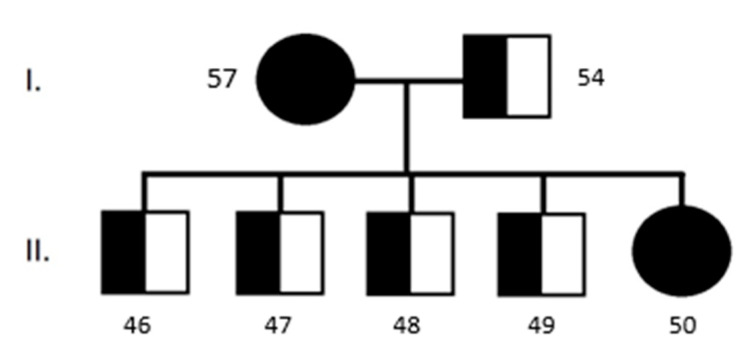
Pedigree schema of analyzed family of Longhaired Collies for CEA (Samples I. 57 and 54; Samples II. 46–50). All samples were confirmed ophthalmologically and genetically.

Some of the clinically healthy Longhaired Collies that were analyzed came from the same family (Figure 3). We can confirm the autosomal recessive characteristics of this disease.

### 3.2. Progressive Retinal Atrophy

Samples for the presence of mutation in gene *PDE6A* causing the RCD3 type of PRA were also analyzed using the same methods of molecular diagnostics. We used a simple PCR method and real-time PCR. We have not confirmed any positive results (Figure 1b) (i.e., genotype aa); our tests indicated that all animals were homozygous wild type (AA). This finding corresponds with clinical findings, according to the age of the dogs and characteristic early expression of the disorder. In affected dogs, we would already observe changes in the ocular background at this age.

## 4. Discussion

The analysis of Collie Eye Anomaly is important in dog breeds with a predisposition to this condition. Collie Eye Anomaly is an inherited ocular disorder characterized by abnormal development of the choroid and sclera, which can lead to visual impairment and, in some cases, blindness. Similarly, as PRA, this abnormality can also be used in human medicine as a suitable model for ocular diseases. CEA in dogs links to possible relevant human traits for microphthalmia and coloboma located on chromosome 2q35. Similarly, PRA in dogs links to a possible relevant human trait for retinitis pigmentosa located on the 5q32 chromosome [1]. Development of a canine genome map has enabled the localization of specific canine disorders with sufficient precision. This knowledge, with the corresponding human map position, can be used to identify the responsible canine locus [18]. In this study, we confirmed the genotype of 16 dogs that had an ophthalmologically confirmed disease. Despite being affected by the disease, these individuals exhibit only mild visual impairment, suggesting a slow progression of the condition. This observation was also confirmed by Palanova in 2015 [19]: only a low number of dogs suffered from defective vision or blindness, but she supposed that they may develop some symptoms in the future. This state is characteristic of CEA, when the degeneration of the ocular tissues is visible only in the first 7 to 12 weeks of life. After this so-called diagnostic window, the pigmentation of the retina starts to develop, and the choroid is not visible anymore. This phenomenon is called “go normal”, and diagnosis of the disease using ophthalmology examination is no longer available [19]. The importance of genetic testing is high, not only because of the possibilities of diagnosis after this period but also because of the high prevalence in other countries. Past studies confirmed a high incidence of CEA in Collies in the Netherlands (40.6%), in Smooth Collies in the United Kingdom (72%), and in Shetland Sheepdogs in Switzerland (15.1%) [20,21,22]. Remarkable high prevalence (83.3%) of the disease was also confirmed in Rough Collies in Thailand [23]. Detection of CEA was also conducted on Australian Shepherd dogs in Australia by Munyard et al. [24]. They found that it was the most common eye disease detected by ophthalmologists in the breed in Australia. Others detected a CEA-affected individual, a homozygous mutation, in an Australian Kelpie dog in Poland [25]. A detection of several eye diseases in Australian Shepherd dogs located in Europe was performed by Majchráková et al. over a 10-year period [26]. Collie Eye Anomaly was detected in 9.71% of tested samples. New studies confirmed the drop in these numbers, which can be associated with the higher interest of breeders in genetic testing and also with the fact that many DNA laboratories currently offer these tests [27]. This trend in reduction was also confirmed by Marelli [11] in predisposed breeds, showing the effectiveness of information campaigns for breeders.

In the case of our testing, we found 15 (55.5%) Longhaired Collies out of 27 that were homozygous recessive (aa) for CEA. However, this finding is somewhat influenced by the low number of samples and affinities between some of the dogs. It is necessary to obtain more samples and data in the future to confirm or refute these findings. We tested dogs from the same parents in the same breeding, who were recessive homozygotes for CEA (aa) and transmitted the diseases to their offspring, even though they were clinically healthy (Figure 3). This confirms how breeding without knowing genotype can result in transmission of the mutation to the offspring. Due to the high prevalence of the CEA in predisposed breeds, affected individuals cannot be fully taken out of the breeding, which will disrupt the gene pool. The best possible variant is to breed a dominant homozygote with a heterozygote. This will lead to a 1:1 ratio of healthy individuals to carriers. Although the frequency of the disease has declined over several years, it is still the second most common congenital eye disease in Australian Shepherds [26]. We also confirmed nine (33.3%) Longhaired Collies to be latent carriers of the disease. These numbers do not correspond to the basic Mendelian type of inheritance, but that can be affected by a lower number of samples.

After testing of the Shetland sheepdog breed, we confirmed the recessive homozygous state of the gene only in one sample (10%) and the heterozygous state of the gene in four samples (40%). The other five samples were dominant homozygotes.

The analysis of the molecular basis of Progressive Retinal Atrophy in dogs, particularly the RCD3 type caused by mutations in the *PDE6A* gene, is important for several reasons. First, these canine models provide valuable insights into the mechanisms underlying inherited retinal degenerative diseases in humans, as many of the genes and pathways involved are conserved across species [28,29]. The *PDE6A*-mutant dog model has been instrumental in advancing our understanding of the role of cyclic GMP dysregulation in photoreceptor cell death and has informed the development of potential therapeutic strategies targeting this pathway [28,30]. Second, the well-characterized nature of the RCD3 condition in dogs and the availability of a large animal model system have made it a valuable tool for testing and evaluating novel treatment approaches, such as gene therapy and neuroprotective therapies. Nearly 100 breeds have an inherited form of PRA. This makes it a good natural animal model for investigation of genetics, disease progression, and possible therapies for both dogs and humans [2]. Finally, the identification of the *PDE6A* mutation in RCD3-affected dogs has enabled the development of genetic testing and screening programs to identify carriers and affected individuals, allowing for informed breeding decisions and the selective elimination of the disease allele from dog populations [30,31,32]. In this study, we did not confirm *PDE6A* mutation in any sample, but this finding does not diminish the importance of testing. Ghilardi in 2023 [33] confirmed that 81.78% of the tested dogs had some type of Progressive Retinal Atrophy. PRA represents a large group of diseases affecting a significant range of dog breeds. It is the most common category of inherited retinal degeneration in dogs [34]. The most significant fact about PRA is the difference in the age of symptom occurrence. With early-onset forms, we can see changes in vision and ocular changes in the first years of age, before adding them to reproduction. However, sometimes the eyesight is not affected to such an extent that its owner notices it. With late-onset forms, the symptoms occur late, and it is possible that these individuals already have offspring. For each type of PRA, a few breeds are most affected; this does not mean that this type cannot occur in other breeds. RCD3 type, which is common for Cardigan Welsh Corgi dogs, was also detected in Chinese Crested dogs [35]. This fact confirms that RCD3 PRA analysis should not be restricted to the predisposed breed and should also be tested in other breeds.

## 5. Conclusions

In conclusion, this study has demonstrated the importance of genetic testing for these diseases to prevent their further spread. Diagnosis should not be restricted to ophthalmological examination, which sometimes cannot be 100% guaranteed. The fact that buccal swabs are also suitable for these techniques, which allow genetic testing without invasive sampling, is also important for future studies of inherited diseases.

## Figures and Tables

**Table 1:** List of used primers with sequence and amplicon size.

Name	Sequence	Expected Amplicon Size
*NHEJ1*-F17	5′-TCTCACAGGCAGAAAGCTCA-3′	636 bp
*NHEJ1*-R17	5′-CCATTCATTCCTTTGCCAGT-3′	
*NHEJ1*-F20	5′-TGGGCTGGTGAACATTTGTA-3′	941 bp
*NHEJ1*-R23	5′-CCTTTTTGTTTGCCCTCAGA-3′	
*NHEJ1*-FW	5′-AGGGTTACCATTTGGGAACTGTCTT-3′	-
*NHEJ1*-RW	5′-AGCTTCTGACAGGCCACAATTATCTA-3′	120 bp
*NHEJ1*-RM	5′-ACCAATCATCATCCAGCCCAGCAGCATTTAA-3′	68 bp
SPJn-F	5′-TCCCATTCAGGTCCCAGAA-3′	212 bp
SPJn-R	5′-TGATGACCTCTGACCTCTG-3′	
SPJm-F	5′-TTCCCATTCAGGTCCCAGAC-3′	213 bp
SPJm-R	5′-GATGACCTCTGACCTCTGAT-3′	

**Table 2:** List of samples analyzed in this study with corresponding genotypes for CEA and RCD3-PRA. Since it is an autosomal recessive disease, the genotypes in this table are identically labeled (AA—dominant homozygote, Aa—heterozygote, and aa—recessive homozygote). Age corresponds with the age of dogs at the time of publishing.

Sample No.	Breed	Gender	Age	Sample Type	CEA RT-PCR	CEA	RCD3-PRA
1.	Australian Shepherd	male	4 years	Buccal swab	Aa	Aa	AA
2.	Australian Shepherd	male	7 years	Buccal swab	AA	AA	AA
3.	Australian Shepherd	female	10 years	Buccal swab	AA	AA	AA
4.	Australian Shepherd	female	3 years	Buccal swab	AA	AA	AA
5.	Australian Shepherd	female	12 years	Blood/EDTA	AA	AA	AA
6.	Australian Shepherd	female	9 years	Blood/EDTA	AA	AA	AA
7.	Australian Shepherd	female	6 years	Blood/EDTA	AA	AA	AA
8.	Australian Shepherd	female	5 years	Blood/EDTA	AA	AA	AA
9.	Australian Shepherd	male	7 years	Blood/EDTA	AA	AA	AA
10.	Australian Shepherd	female	3 years	Blood/EDTA	AA	AA	AA
11.	Australian Shepherd	female	3 years	Buccal swab	AA	AA	AA
12.	Australian Shepherd	male	4 years	Buccal swab	AA	AA	AA
13.	Australian Shepherd	female	3 years	Buccal swab	AA	AA	AA
14.	Australian Shepherd	female	6 years	Buccal swab	AA	AA	AA
15.	Australian Shepherd	male	7 years	Buccal swab	AA	AA	AA
16.	Australian Shepherd	female	8 years	Buccal swab	Aa	Aa	AA
17.	Australian Shepherd	male	13 years	Buccal swab	AA	AA	AA
18.	Australian Shepherd	male	3 years	Buccal swab	AA	AA	AA
19.	Australian Shepherd	male	7 years	Buccal swab	AA	AA	AA
20.	Australian Shepherd	male	4 years	Buccal swab	AA	AA	AA
21.	Australian Shepherd	male	4 years	Buccal swab	AA	AA	AA
22.	Australian Shepherd	female	6 years	Buccal swab	AA	AA	AA
23.	Australian Shepherd	male	2 years	Blood/EDTA	AA	AA	AA
24.	Australian Shepherd	female	2 years	Blood/EDTA	AA	AA	AA
25.	Australian Shepherd	female	2 years	Blood/EDTA	AA	AA	AA
26.	Australian Shepherd	female	6 years	Blood/EDTA	AA	AA	AA
27.	Australian Shepherd	female	2 years	Blood/EDTA	AA	AA	AA
28.	Australian Shepherd	female	2 years	Blood/EDTA	AA	AA	AA
29.	Australian Shepherd	female	3 years	Buccal swab	AA	AA	AA
30.	Border Collie	male	8 years	Buccal swab	AA	AA	AA
31.	Border Collie	female	7 years	Buccal swab	AA	AA	AA
32.	Border Collie	male	4 years	Buccal swab	AA	AA	AA
33.	Border Collie	female	3 years	Buccal swab	AA	AA	AA
34.	Border Collie	male	7 years	Buccal swab	AA	AA	AA
35.	Border Collie	female	7 years	Buccal swab	Aa	Aa	AA
36.	Border Collie	female	3 years	Buccal swab	AA	AA	AA
37.	Border Collie (mixed)	male	3 years	Buccal swab	AA	AA	AA
38.	Border Collie (mixed)	male	6 years	Buccal swab	AA	AA	AA
39.	Longhaired Collie	female	6 years	Buccal swab	aa	aa	AA
40.	Longhaired Collie	male	4 years	Buccal swab	aa	aa	AA
41.	Longhaired Collie	male	10 years	Buccal swab	aa	aa	AA
42.	Longhaired Collie	female	8 years	Buccal swab	aa	aa	AA
43.	Longhaired Collie	female	5 years	Buccal swab	aa	aa	AA
44.	Longhaired Collie	male	4 years	Buccal swab	aa	aa	AA
45.	Longhaired Collie	female	6 years	Blood/EDTA	aa	aa	AA
46.	Longhaired Collie	female	1 years	Buccal swab	aa	aa	AA
47.	Longhaired Collie	male	1 years	Buccal swab	Aa	Aa	AA
48.	Longhaired Collie	male	1 years	Buccal swab	Aa	Aa	AA
49.	Longhaired Collie	male	1 years	Buccal swab	Aa	Aa	AA
50.	Longhaired Collie	male	1 years	Buccal swab	Aa	Aa	AA
51.	Longhaired Collie	female	11 years	Buccal swab	aa	aa	AA
52.	Longhaired Collie	female	8 years	Buccal swab	AA	AA	AA
53.	Longhaired Collie	female	1 years	Buccal swab	AA	AA	AA
54.	Longhaired Collie	male	4 years	Buccal swab	Aa	Aa	AA
55.	Longhaired Collie	male	4 years	Buccal swab	Aa	Aa	AA
56.	Longhaired Collie	male	4 years	Buccal swab	Aa	Aa	AA
57.	Longhaired Collie	female	4 years	Buccal swab	AA	AA	AA
58.	Longhaired Collie	female	1 years	Buccal swab	aa	aa	AA
59.	Longhaired Collie	male	1 years	Buccal swab	Aa	Aa	AA
60.	Longhaired Collie	male	1 years	Buccal swab	aa	aa	AA
61.	Longhaired Collie	male	1 years	Buccal swab	aa	aa	AA
62.	Longhaired Collie	male	3 years	Buccal swab	aa	aa	AA
63.	Longhaired Collie	female	7 years	Buccal swab	aa	aa	AA
64.	Longhaired Collie	female	3 years	Buccal swab	Aa	Aa	AA
65.	Longhaired Collie	female	2 years	Buccal swab	aa	aa	AA
66.	Shetland Sheepdog	female	9 years	Buccal swab	Aa	Aa	AA
67.	Shetland Sheepdog	female	12 years	Buccal swab	AA	AA	AA
68.	Shetland Sheepdog	male	10 years	Buccal swab	AA	AA	AA
69.	Shetland Sheepdog	female	2 years	Buccal swab	Aa	Aa	AA
70.	Shetland Sheepdog	female	6 years	Buccal swab	AA	AA	AA
71.	Shetland Sheepdog	female	3 years	Buccal swab	AA	AA	AA
72.	Shetland Sheepdog	female	4 years	Blood/EDTA	Aa	Aa	AA
73.	Shetland Sheepdog	male	3 years	Blood/EDTA	AA	AA	AA
74.	Shetland Sheepdog	male	5 years	Buccal swab	Aa	Aa	AA
75.	Shetland Sheepdog	female	5 years	Buccal swab	aa	aa	AA

## Data Availability

The data presented in this study are available on request from the corresponding author due to ethical reasons.
